# On Infection from Inoculated Small Pox

**Published:** 1811-06

**Authors:** 


*To the Editors of the Medical and Physical Journal.*

Gentlemen,

Your Correspondent, Mr. Barlow, at p. 390 of your last Number, doubts the fact of the inoculated Small-pox proving infectious, except under particular circumstances, and then rarely; he is aware that this sentiment will meet with an adverse opinion from the *advocates of vaccination*, yet he is actuated to hazard it—*from motives of utility!*

By mentioning the *advocates of vaccination* in particular, does your correspondent mean to insinuate, that *they* only will differ in opinion with him on the subject? I thought this matter had been decided long before either *vaccinists* or *anti-vaccinists* were known. By merely turning to your 14th volume, page 311, your Correspondent may be made acquainted with several fatal cases of small-pox communicated by casual infection from those who had been inoculated; among which is the never-to-be-forgotten fact, recorded by Dr. Willan, in the year 1796, (before vaccination was in use) of the inoculation of a child, whose parents kept a shop in a small court. *From this child seventeen persons caught the small-pox in the natural way, and eight of them died.* Thus in order to save the life of one person, seventeen were put in danger of their lives, and eight, or nearly one half, were actually sacrificed.

These cases are comparatively of modern date; perhaps your Correspondent will be better satisfied with some of an earlier period. Let us go back to the year 1721, and we shall find records to substantiate the assertion that "infection may be traced from inoculation *even when the inoculated disease has not assumed the confluent character.*"

Dr. William Douglas "a physician of the best credit and practice," at Boston in New England, writing to Dr. Alexander Stuart, under date of December 20, 1721,* says, "at first they gave out that it was a *method not infecting*, procuring only a small quantity of eruptions, but never death, nor any bad consequence, and was an infallible security against ever after having the small-pox."

"*We soon found it infecting; many have died of the infection received from the inoculated*, whose deaths in a great

* Published by Dr. Wagstaffe as an Appendix to his "Letter to Dr. Freind, shewing the danger and uncertainty of inoculating the Small Pox." 2d Edit. 1722.

measure measure lie at the inoculators' doors. *Then the persons gave up this point*, but continued to maintain the rest, *till they found some of the inoculated with immense numbers of pustules*, &c. &c.

These extracts confirm the fact, which Mr. Barlow doubts. *The small-pox*, it appears, *was propagated by infection from the inoculated, when the disease was not confluent;* for the *immense number of pustules*, or, in other words, the confluent small-pox from inoculation, was not known till after the idle notion of the non-infectious nature of the inoculated small-pox *had been given up*.

It is very well known, that Mr. Maitland, the first English inoculator, entertained the opinion, that the inoculated small-pox would not communicate the disease by casual infection; and in consequence, he took no care to separate his inoculated patients from persons liable to receive the infection; but woeful experience soon taught him otherwise, and made him more cautious in future.

If the pages of your Journal are not better occupied, I will request you to insert an extract from another letter of the same Doctor Douglass. It shews, in a very lively manner, the danger of promiscuous inoculation, and will, I think, convince all who consider the matter temperately, that the Governors of the Small-pox Hospital, and of some other charitable Institutions, have acted a wise, humane, and patriotic part by altogether prohibiting variolous inoculation at these institutions. I wish the facts stated by the worthy Doctor, who "though dead, yet speaketh," may have the effect of influencing others to refrain from sowing the seeds of so destructive a malady as the Small-pox, in the narrow courts and alleys of this great metropolis; where, unfortunately, a soil is found too well fitted for the propagation of this loathsome and fatal disease: but I can have little expectation that this or any thing else will induce those persons to desist from the practice, *who find their account, in being at the expence of advertising for poor objects to be inoculated gratuitously*.

There are, I know, in this town, apothecaries who profess to *give advice gratis*, at certain hours of the day, but oblige their patients to pay for their medicines; and I apprehend that the same plan is followed by the advertisers of small-pox inoculation *gratis*. If so, it may certainly answer their purpose; for what with preparing persons for the inoculation, purging them afterwards, and giving them medicines during the disease, a large expence may be incurred under the idea of performing an act of humanity and kindness *gratis*.

Extract of a letter from Dr. William Douglass, to Dr. Alexander Stuart.

*Boston, Feb.* 15, 1722.

"Since my last to you the small-pox has made little or no progress in the country. Our newspapers tell us that in some towns it is entirely ceased, in others abated. Who then but madmen would have advised inoculation in the severest season to those who are likely for ever to escape the small-pox? In this town several hundreds have escaped, and it is probable many more might have escaped, (as was the case nineteen years ago) *if inoculation had not rendered the infection so universal and intense.*

"Last small-pox, *the month of the greatest mortality* (December, a severe winter month) *did not exceed eighty persons*: at this time, *the month of the greatest mortality* (October, a favourable autumn month) *exceeded four hundred burials*, which is more than all that died of the small-pox nineteen years ago.

"For the three months of September, October, and November last, *in which inoculation prevailed*, the town was a mere hospital, and *we buried seven hundred and sixty persons*. The last small-pox spread gradually in the extent of ten or a dozen months, and vast numbers escaped. *Inoculation of the small-pox this time set us all in a flame, and in half the time leaves few exempt from its rage.* With what face can any man call our methods of inoculation a regular procedure?

"I heartily wish success to this and all means designed to alleviate the epidemic distempers incident to mankind, whether casually discovered, or ingeniously contrived by the sons of Æsculapius; but rashness and headstrong irregular procedure I shall for ever exclaim against, ESPECIALLY THAT DETESTABLE WICKEDNESS OF SPREADING THE INFECTION." I am, &c.

REPREHENSOR.

*May* 7, 1811.

